# Evidence-Based Management and Functional Outcomes of Femoral Head Avascular Necrosis: A Systematic Review

**DOI:** 10.7759/cureus.114825

**Published:** 2026-08-20

**Authors:** Sakshant Desai, Sandeep Shinde, Ruturaj Desai, Makarand Mane, Anjali Shinde

**Affiliations:** 1 Department of Musculoskeletal Sciences, Krishna College of Physiotherapy, Krishna Vishwa Vidyapeeth (Deemed to be University), Karad, IND; 2 Department of Pharmacology, Maharashtra Institute of Medical Education and Research, Pune, IND; 3 Department of Medicine, Krishna Vishwa Vidyapeeth (Deemed to be University), Karad, IND; 4 Department of Pharmacology, Krishna Vishwa Vidyapeeth (Deemed to be University), Karad, IND

**Keywords:** avascular necrosis, core decompression, extracorporeal shock wave therapy, functional outcome, osteonecrosis of the femoral head, platelet-rich plasma, stem cell therapy, systematic review, total hip arthroplasty

## Abstract

Avascular necrosis of the femoral head (AVNFH), also known as osteonecrosis of the femoral head, is a progressive musculoskeletal disorder caused by compromised blood supply to the femoral head, causing osteocyte death, structural collapse, pain, and functional disability. Despite advances in diagnostic imaging and treatment modalities, optimal management remains challenging because of multifactorial etiology and variable clinical presentation of the disease.

To systematically evaluate the etiopathogenesis, evidence-based management strategies, and functional outcomes associated with AVNFH.

A systematic review was conducted in compliance with Preferred Reporting Items for Systematic Reviews and Meta-Analyses (PRISMA) 2020 guidelines. Electronic literature searches identified studies investigating etiology, management, and functional outcomes of AVNFH. Adult patients with traumatic or non-traumatic AVNFH across pre-collapse and post-collapse stages were included. Randomized controlled trials and observational studies evaluating surgical, biological, pharmacological, physical therapy, or arthroplasty interventions and reporting validated clinical outcomes were eligible. Methodological quality was assessed using the Cochrane Risk of Bias 2 tool for randomized controlled trials and the Newcastle-Ottawa Scale for observational studies.

Twenty-two studies published between 2010 and 2023 met the eligibility criteria and were included in the review. Evidence demonstrated that corticosteroid exposure, alcohol consumption, trauma, and idiopathic factors were the main contributors to disease development. Joint-preserving procedures, particularly core decompression combined with biological augmentation techniques such as platelet-rich plasma, bone marrow-derived cellular therapies, and stem-cell-based interventions, consistently demonstrated improved functional outcomes and delayed disease progression. Vascularized grafting procedures enhanced femoral head vascularity and survivorship, while extracorporeal shock wave therapy and hyperbaric oxygen therapy provided clinically meaningful improvements in pain and function in early-stage disease. Pharmacological interventions showed variable effectiveness, with zoledronate failing to significantly prevent femoral head collapse. In advanced disease, bipolar hip arthroplasty and cementless total hip arthroplasty produced substantial functional improvement and reliable symptom relief. Risk-of-bias assessment indicated predominantly moderate methodological quality, with heterogeneity observed across study designs, interventions, and outcome measures.

AVNFH is a multifactorial condition in which early diagnosis and timely intervention are critical for preserving hip function and delaying disease progression. Current evidence supports stage-specific management, with biologically augmented joint-preserving procedures demonstrating most favorable outcomes in pre-collapse disease, while arthroplasty remains definitive treatment for advanced femoral head collapse. Although regenerative therapies show considerable promise, further high-quality multicenter randomized controlled trials with standardized outcome measures and long-term follow-up are required to establish optimal treatment algorithms and strengthen the evidence base for clinical decision-making.

## Introduction and background

Osteonecrosis of the femoral head (ONFH), also known as avascular necrosis of the femoral head (AVNFH), is a progressive disorder characterized by ischemic death of osteocytes and subchondral bone marrow. The condition eventually leads to structural collapse of the femoral head if left untreated [1]. Often termed the *coronary disease of the hip*, this mechanically destructive condition affects younger, highly active adults (primarily in the third to fifth decades of life) and accounts for approximately 10% to 12% of all total hip arthroplasties (THAs) performed annually [2]. The pathogenesis of ONFH represents the final common pathway of subchondral microcirculatory failure driven by three primary mechanisms: direct vascular interruption (e.g., displaced femoral neck fractures), intravascular thrombotic occlusion (e.g., hypercoagulability or sickle cell hemoglobinopathies), and extravascular compression [3]. Atraumatic etiologies are predominantly associated with high-dose glucocorticoid administration and chronic alcohol abuse. These specific biochemical insults precipitate accelerated adipogenesis, expansion of marrow lipocytes that acutely elevate intraosseous pressure, induce endothelial cell apoptosis, and subsequently initiate local ischemia [3,4]. While magnetic resonance imaging (MRI) provides the non-invasive clinical standard for staging pre-collapse lesions via classification systems such as Association Research Circulation Osseous (ARCO) or Ficat-Arlet systems, reliance solely on clinicoradiological findings is subject to critical diagnostic error [4-6]. Retrospective histopathological evaluation of non-fractured THA specimens demonstrates that up to 31% of histologically confirmed ONFH cases are preoperatively misdiagnosed as primary osteoarthritis, particularly among older patients without classical risk factors [7]. An accurate diagnosis in atypical presentations requires a thorough pathological examination to differentiate ONFH from isolated degenerative joint disease [8]. If left untreated, up to 80% of untreated asymptomatic lesions exhibit a highly predictable trajectory toward subchondral fracture, irreversible articular collapse, and profound physical disability within two years [9]. AVNFH predominantly affects young and middle-aged adults during their most productive years, resulting in chronic pain, progressive functional limitation, reduced mobility, and substantial impairment in quality of life. Without timely diagnosis and appropriate intervention, progressive collapse of the femoral head frequently necessitates THA, thereby increasing both the individual disability burden and healthcare costs. Consequently, early recognition and effective stage-specific management are essential to preserve hip function and delay disease progression [1,3,8]. Previous reviews have evaluated individual aspects of AVNFH, including disease pathophysiology, pharmacological management, conservative treatment, and specific surgical interventions. However, many have focused on individual therapeutic modalities rather than providing a comprehensive synthesis of the etiopathogenesis, evidence-based management, and functional outcomes across both operative and nonoperative approaches. Furthermore, recent advances have expanded the therapeutic landscape, highlighting the need for an updated systematic review that critically evaluates the current evidence and identifies areas requiring further investigation [3,8]. In strict alignment with the Preferred Reporting Items for Systematic Reviews and Meta-Analyses (PRISMA) 2020 framework, the objective of this systematic review is to synthesize the current clinical evidence base detailing the etiopathogenesis of AVNFH, critically evaluate joint-preserving versus arthroplasty interventions, and objectively quantify the functional outcomes of different therapeutic approaches.

Evidence-based management and functional outcomes

Surgical

Pre-collapse surgical intervention aims to effectively decompress elevated intraosseous pressure and stimulate regional neovascularization. Core decompression (CD) remains the primary first-line joint-preserving procedure [9]. Contemporary surgical approaches favor multiple small-diameter drilling techniques over traditional single large-diameter drilling techniques to reduce the risk of iatrogenic subtrochanteric fracture while achieving equivalent necrotic clearance [9,10]. To structurally bridge the void left by decompression, vascularized and non-vascularized fibular autografts are frequently integrated to provide an osteoconductive and osteoinductive scaffold, successfully preserving femoral head sphericity in small-to-medium Ficat Stage I and II lesions [10]. Conversely, the insertion of isolated porous tantalum rods for structural support has demonstrated catastrophic clinical outcomes; Mid-term follow-up studies have revealed failure rates approaching 56% at 1.45 years, presenting no clinical superiority over standard CD and severely complicating subsequent conversion to arthroplasty [11]. Joint-preserving proximal femoral osteotomies offload mechanical stress from the infarcted articular dome to healthy cartilage, though success relies heavily on precise indications and strict geometric parameters [11,12].

Pharmacological

Medical management targets the underlying biochemical mechanisms of ischemia before subchondral collapse, although its overall efficacy remains controversial. Lipid-lowering agents, primarily statins, reduce corticosteroid-induced adipocyte hypertrophy, thereby reducing extravascular marrow compression. Anticoagulant therapy with enoxaparin effectively treats thrombophilic occlusion, thereby slowing the progression of early-stage lesions [12]. Iloprost, a synthetic prostacyclin analog administered intravenously, induces systemic vasodilation and reduces capillary permeability, resulting in rapid reduction of bone marrow edema, pain relief, and short-term functional improvement [13]. Bisphosphonates (e.g., alendronate) are used to inhibit osteoclast-mediated bone resorption, thereby preserving the mechanical integrity of the necrotic trabeculae during the revascularization phase [13]. However, systematic reviews of randomized controlled trials have demonstrated considerable heterogeneity in clinical efficacy, indicating that bisphosphonates do not consistently prevent femoral head collapse or eliminate the need for eventual arthroplasty [14].

Physical Therapies

Non-invasive biophysical modalities use targeted energy fields to stimulate localized tissue regeneration and serve as important joint-preserving adjuncts. Extracorporeal shockwave therapy (ESWT) mechanically enhances the expression of vascular endothelial growth factor (VEGF) and bone morphogenetic proteins (BMPs), thereby promoting angiogenesis and osteoblast proliferation. Longitudinal clinical studies have suggested that ESWT may provide clinical outcomes comparable to those of surgical CD in selected patients with early-stage disease, although further high-quality evidence is required to confirm its comparative effectiveness [14,15]. Contemporary management of AVNFH is guided by disease staging systems such as the ARCO classification, which assists clinicians in selecting appropriate treatment strategies according to disease severity. Early-stage disease is generally managed using joint-preserving procedures, whereas advanced stages commonly require THA. Pulsed electromagnetic field (PEMF) therapy provides continuous electrical stimulation to preserve structural integrity, while hyperbaric oxygen therapy increases extracellular oxygen tension to reverse osteocyte hypoxia and reduce intraosseous edema [15]. Individual case reports have described improvements following physiotherapy interventions; however, higher quality evidence evaluating rehabilitation strategies in AVNFH remains limited [16,17].

Arthroplasty

The onset of macroscopic subchondral collapse and secondary osteoarthritis (ARCO Stage III/IV) generally precludes joint-preserving procedures, making THA the definitive treatment option [17]. Although THA was historically associated with premature implant failure and accelerated wear rates in younger, high-demand patients with AVNFH, the introduction of highly cross-linked polyethylene and modern cementless stem designs has significantly improved implant survival [8]. Contemporary THA provides substantial pain relief and rapid restoration of functional mobility, with long-term implant survival comparable to or better than that reported for patients undergoing THA for primary osteoarthritis [18].

Others

Advanced cellular therapies are increasingly used as regenerative adjuncts to CD. Intraosseous administration of autologous mesenchymal stem cells (MSCs), harvested through bone marrow aspirate concentrate (BMAC), is intended to enhance bone regeneration within the necrotic trabecular framework, significantly delaying disease progression and improving radiological outcomes compared with CD alone [19]. Within complementary medicine, ultrasound-guided acupotomy, a minimally invasive needle-knife procedure, is used to release contracted periarticular tissues and the joint capsule [20]. When combined with targeted herbal therapies, randomized controlled trials have shown that acupotomy significantly improves the Harris Hip Score (HHS) and visual analog scale (VAS) pain scores without the morbidity associated with traditional open surgery [20,21,22]. The Western Ontario and McMaster Universities Osteoarthritis Index (WOMAC) is a validated patient-reported outcome measure used to assess pain, stiffness, and physical function in individuals with hip and knee osteoarthritis [23]. The Lequesne Algofunctional Index is a validated assessment tool used to evaluate the severity of osteoarthritis and its functional impact, particularly in the hip and knee [24]. These assessment tools provide standardized measures of pain, functional limitation, and disease severity and are commonly used to evaluate clinical outcomes in osteoarthritis. The pathogenesis of glucocorticoid-induced AVNFH involves endothelial dysfunction, impaired angiogenesis, osteocyte apoptosis, and disruption of the femoral head microcirculation, leading to ischemia and progressive bone necrosis [25].

Despite the availability of numerous operative and nonoperative treatment options, the optimal management of AVNFH remains controversial. Clinical decision-making is influenced by disease stage, lesion size, etiology, patient age, and functional demands, while considerable variability exists in surgical techniques, regenerative therapies, rehabilitation protocols, and reported outcome measures. These factors contribute to inconsistent clinical outcomes and make direct comparison between treatment strategies challenging [3,8,9,12-14].

## Review

Methodology

Study Design

This systematic review was conducted in accordance with the PRISMA 2020 guidelines [26]. The review protocol was prospectively registered with the International Prospective Register of Systematic Reviews (PROSPERO) (Registration No. CRD420261455853). The review was conducted in accordance with the registered protocol to ensure methodological rigor, transparency, and reproducibility in evaluating the etiopathogenesis, evidence-based management, and functional outcomes of AVNFH.

Search Strategy

This systematic analysis evaluates current protocols for identifying and curating femoral head avascular necrosis (AVN), prioritizing studies that provide a strong evidentiary basis for clinical practice. A comprehensive literature search was conducted in PubMed, Scopus, Web of Science, Cochrane Library, and Google Scholar from database inception to June 2026. Medical Subject Headings (MeSH) terms and free-text keywords such as “Osteonecrosis,” “Avascular Necrosis,” “Femoral Head,” “Core Decompression,” “Stem Cells,” “Platelet Rich Plasma,” and “Arthroplasty” were combined using Boolean operators.

Study Selection and Data Extraction

Following the PRISMA 2020 guidelines and the predefined inclusion and exclusion criteria, two reviewers independently screened the titles, abstracts, and full-text articles for eligibility. Any discrepancies between the reviewers were resolved through discussion and consensus. If consensus could not be reached, a third reviewer adjudicated the final decision.

Data Synthesis

Due to the substantial clinical and methodological heterogeneity among the included studies, including differences in study design, participant characteristics, interventions, outcome measures, and duration of follow-up, a quantitative meta-analysis was not considered appropriate. Therefore, a qualitative narrative synthesis was performed in accordance with the PRISMA 2020 reporting guidelines. The included studies were systematically grouped according to their primary focus, including etiopathogenesis, joint-preserving surgical procedures, regenerative therapies, pharmacological interventions, physical therapy, and arthroplasty. The findings were synthesized by comparing the available evidence regarding treatment effectiveness, functional outcomes, and methodological quality across studies. This approach enabled a comprehensive interpretation of the evidence while acknowledging the heterogeneity of the included studies.

Inclusion Criteria

We included English-language studies featuring adult human participants with a confirmed diagnosis of symptomatic traumatic or non-traumatic AVNFH across both pre-collapse and post-collapse disease stages. Randomized controlled trials, prospective and retrospective cohort studies, comparative studies, epidemiological studies, and case series were included because the objective of this review was to comprehensively evaluate the etiopathogenesis, evidence-based management strategies, and functional outcomes of AVNFH. Randomized controlled trials provide the highest level of evidence for treatment effectiveness; however, they are limited for evaluating uncommon interventions, long-term outcomes, prognostic factors, and rare clinical presentations. Therefore, observational studies and case series were included to provide complementary clinical evidence and a broader understanding of disease characteristics and treatment outcomes. Because of the anticipated clinical and methodological heterogeneity across study designs, a qualitative evidence synthesis was performed rather than a quantitative meta-analysis.

Exclusion Criteria

Exclusion criteria consisted of non-peer-reviewed evidence, incomplete case reports, and studies lacking definitive clinical endpoints or sufficient longitudinal follow-up. We also excluded patients exhibiting confounding localized pathologies such as active joint infections, neoplastic processes, or acute pathologic fractures, alongside individuals presenting severe systemic comorbidities like advanced cardio-cerebrovascular disease, profound renal dysfunction, immunosuppression, or active pregnancy. To ensure data integrity, hips previously subjected to invasive surgical interference or metallic hardware implantation were removed to prevent etiological contamination.

Quality Assessment

Methodological quality and the risk of bias were appraised using study-specific validated tools to accommodate the structural heterogeneity of the included study designs. Randomized controlled trials were assessed using the Cochrane Risk of Bias 2 (RoB 2) tool to scrutinize critical domains such as randomization sequence generation, allocation concealment, and blinding integrity, while retrospective and prospective observational cohorts were assessed using the Newcastle-Ottawa Scale (NOS) to validate patient selection, cohort comparability, and follow-up adequacy [27,28].

Results

**Figure 1:**
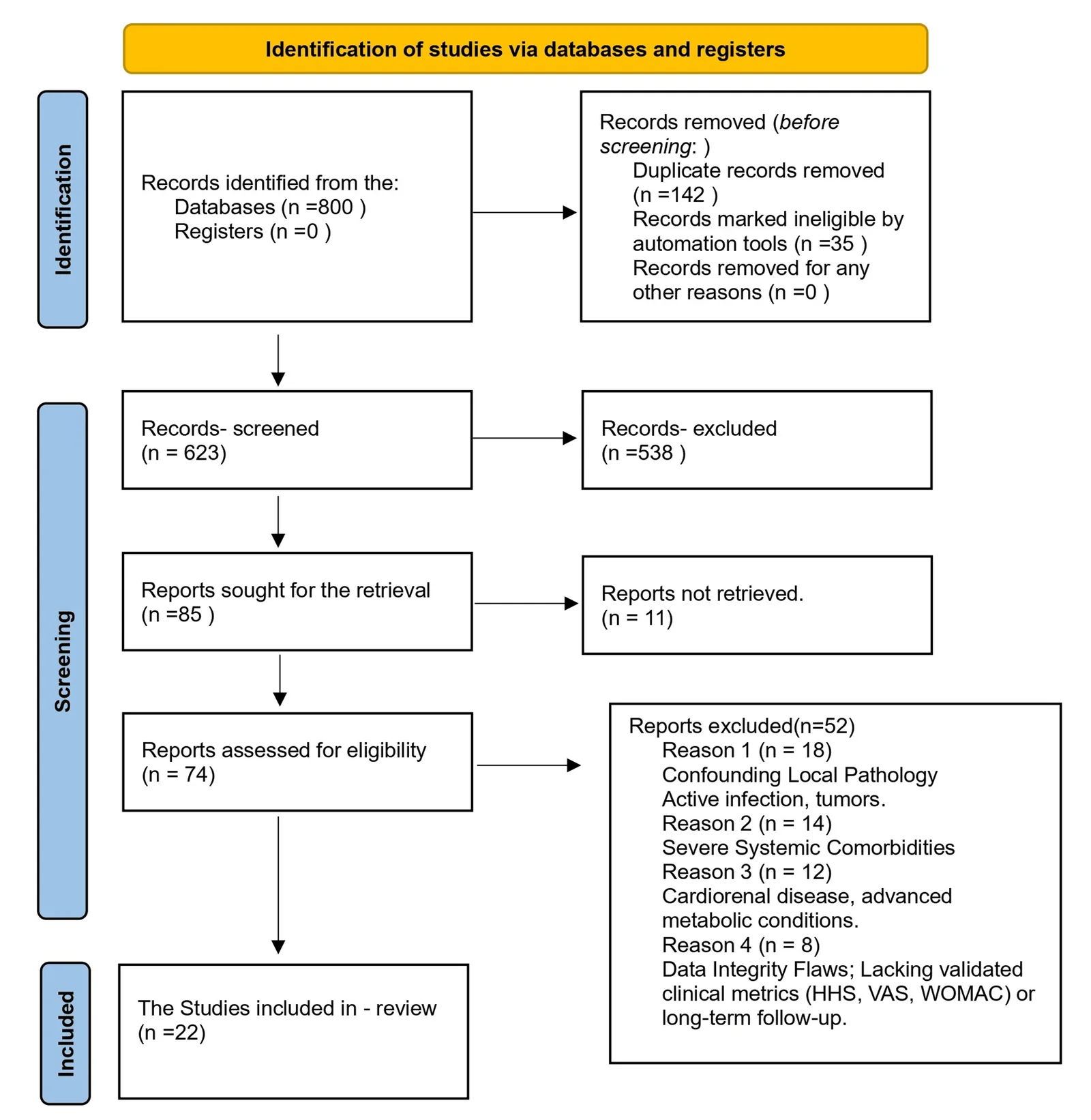
PRISMA flowchart. PRISMA, Preferred Reporting Items for Systematic Reviews and Meta-Analyses; HHS, Harris Hip Score; VAS, visual analog scale; WOMAC, Western Ontario and McMaster Universities Osteoarthritis Index

A total of 800 database records were identified, and 0 were from registers. Before screening, 142 duplicates and 35 automated exclusions were removed, leaving 623 records for title and abstract screening. Of these, 538 records were excluded. Out of the 85 reports sought for retrieval, 11 could not be retrieved. The remaining 74 full-text reports, assessed for eligibility. A total of 52 reports were excluded due to data integrity flaws/lacking validated clinical metrics (*n* = 8), confounding local pathology (*n* = 18), severe systemic comorbidities (*n* = 14), and cardiorenal or advanced metabolic conditions (*n* = 12). Ultimately, 22 studies met all criteria and were included in the PRISMA flowchart (Figure 1) [26].

The details of the 22 included articles were summarized. The studies were published between 2010 and 2026 and comprised randomized controlled trials, retrospective cohort studies, comparative studies, case series, and epidemiological investigations, with sample sizes ranging from 20 to 249 participants.

Etiopathogenesis

Two studies evaluated the etiopathogenesis of ONFH. Vardhan et al. investigated the epidemiological profile and associated risk factors, whereas Lin et al. assessed prognostic factors related to femoral head collapse [29,30].

Joint-Preserving Surgical Procedures

Six studies evaluated joint-preserving surgical procedures. Ma et al., Elmalı et al., Sun et al., Li et al., Cao et al., and Kawano et al. investigated core decompression, vascularized bone grafting, impacted bone grafting, and rotational osteotomy [31-36].

Regenerative Therapies

Seven studies investigated regenerative therapies. Mao et al., Pepke et al., Talathi and Kamath, Xian et al., Aggarwal et al., Hauzeur et al., and Nally et al. evaluated bone marrow-derived therapies, stem-cell therapies, platelet-rich plasma, recombinant human bone morphogenetic protein-2, and osteoblastic cell therapy [37-43].

Pharmacological Interventions

Two studies evaluated pharmacological interventions. Lee et al. investigated zoledronate therapy, while Liyou et al. evaluated intravenous Cervus and Cucumis polypeptides [44,45].

Physical Therapies

Three studies evaluated physical therapy interventions. Camporesi et al. investigated hyperbaric oxygen therapy, whereas Wang et al. and Zhu et al. evaluated extracorporeal shock wave therapy [46-48].

Arthroplasty

Two studies evaluated arthroplasty procedures. Dudani et al. investigated bipolar hip arthroplasty, while Gao et al. evaluated cementless THA. The findings highlight the importance of stage-specific management and the growing role of regenerative therapies in improving clinical outcomes, as given in Table 1 [49,50].

**Table 1:** Baseline attributes and key findings of the included studies. RCT, randomized controlled trial; ROM, range of motion; HBOT, hyperbaric oxygen therapy; ONFH, osteonecrosis of the femoral head; WOMAC, Western Ontario and McMaster Universities Osteoarthritis Index; HHS, Harris Hip Score; rhBMP-2, recombinant human bone morphogenetic protein-2; ARCO, Association Research Circulation Osseous; PBSC, peripheral blood stem cells; THA, total hip arthroplasty; ESWT, extracorporeal shock wave therapy; CD, core decompression; VAS, visual analog scale; MRI, magnetic resonance imaging; SPECT, single-photon emission computed tomography; CT, computed tomography; CDBG, core decompression bone graft; ARO, anterior rotational osteotomy; PRP, platelet-rich plasma; MSR, medial space ratio

Author(s)	Year	Study design	Sample size	Functional outcome measure	Pathogenesis treatment	Result	Conclusion
Vardhan et al. [29]	2018	Retrospective Epidemiological Study	249 patients (382 hips)	HHS, ARCO Stage	Epidemiological analysis	Steroid use was the most common associated factor (37.3%).	ONFH predominantly affects young males and is strongly associated with steroid exposure.
Lin et al. [30]	2021	Retrospective Prognostic Study	135 patients (151 hips)	Collapse Rate, Survival Analysis	Medial Space Ratio assessment	MSR >20.35 was associated with significantly increased collapse risk.	MSR may serve as a predictor of femoral head collapse.
Ma et al. [31]	2014	Prospective Double-Blind RCT	45 patients (53 hips)	Lequesne Index, WOMAC	Core decompression + autologous bone marrow buffy coat grafting	Lower progression rate and superior symptom improvement compared with controls.	Bone marrow buffy coat grafting enhanced the effectiveness of core decompression.
Elmalı et al. [32]	2014	Retrospective Case Series	22 patients (26 hips)	Harris Hip Score (HHS)	Core decompression + vascularized pedicled iliac bone graft	Mean HHS improved from 52 to 82.8 with 69% good-to-excellent outcomes.	Vascularized iliac grafting produced favorable outcomes in early-stage disease.
Sun et al. [33]	2014	Comparative Cohort Study	42 patients (72 hips)	Harris Hip Score	Impacted bone grafting ± rhBMP-2	Survival rate was higher in the rhBMP-2 group (81.8% vs. 71.8%).	rhBMP-2 may enhance bone repair and improve clinical outcomes.
Li et al. [34]	2016	Randomized Controlled Trial	39 patients (47 hips)	Harris Hip Score	Core decompression vs. quadratus femoris muscle - pedicle grafting	Both groups improved similarly; CD had less operative trauma.	Core decompression remains a simpler and effective treatment option.
Cao et al. [35]	2017	Randomized Clinical Trial	27 patients (54 hips)	Harris Hip Score, SPECT/CT Vascularity	Free vascularized fibular grafting vs. core decompression	Vascularized grafting improved vascularity and hip survival.	Fibular grafting was superior to decompression alone.
Kawano et al. [36]	2018	Retrospective Comparative Study	95 hips	PROMs, Hip Survival	Anterior rotational osteotomy	Ten-year hip survival reached 85.4%.	ARO is an effective long-term joint-preserving procedure.
Mao et al. [37]	2015	Randomized Controlled Trial	55 patients (89 hips)	Harris Hip Score, ARCO Stage, THA rate	Mechanical support + intra-arterial PBSC infusion	THA requirement was significantly lower in the stem-cell group (6.3% vs. 22%).	PBSC therapy improved hip survival and delayed progression.
Pepke et al. [38]	2016	Prospective Randomized Trial	24 patients	HHS, VAS, MRI	Core decompression + bone marrow concentrate	No significant advantage over core decompression alone.	Additional bone marrow concentrate showed limited short-term benefit.
Talathi and Kamath [39]	2018	Retrospective Case Series	43 hips (28 patients)	VAS, Radiographic Progression	Core decompression + autologous stem-cell implantation	VAS improved from 7.8 to 2.5; only 3 hips progressed.	Stem-cell implantation may delay collapse and improve symptoms.
Xian et al. [40]	2019	Comparative Clinical Study	46 patients	HHS, VAS	PRP-incorporated autologous bone grafting	Better clinical success, radiological outcomes, and hip survival.	PRP augmentation improved outcomes in precollapse ONFH.
Aggarwal et al. [41]	2020	Prospective Double-Blind Randomized Study	40 patients (53 hips)	HHS, MRI, Pain Score	Core decompression + PRP	Improved pain, function, and delayed disease progression.	PRP enhanced the effectiveness of core decompression.
Hauzeur et al. [42]	2020	Randomized Controlled Trial	56 patients	Functional assessment, Imaging	Osteoblastic cell therapy	Safe treatment with modest clinical benefit.	Cell therapy showed potential but requires further investigation.
Nally et al. [43]	2017	Comparative Retrospective Study	97 hips	THA Conversion Rate	CD vs. CDBG vs. Stem Cells	No significant difference in THA conversion among groups.	Biological augmentation did not significantly alter long-term survival.
Lee et al. [44]	2015	Multicenter Randomized Trial	110 hips	HHS, WOMAC, Collapse Rate	Zoledronate therapy	No significant reduction in collapse or THA conversion.	Zoledronate did not prevent disease progression.
Liyou et al. [45]	2016	Randomized Clinical Trial	96 patients	HHS, VAS	Intravenous Cervus and Cucumis polypeptides	Greater improvement in pain and function than controls.	The therapy appeared safe and clinically beneficial.
Camporesi et al. [46]	2010	Prospective Double-Blind RCT	20 patients	Pain score, ROM, Stabilometry	Hyperbaric oxygen therapy (HBOT)	Significant pain reduction and functional improvement; no patient required arthroplasty at seven years.	HBOT may be effective in early-stage ONFH and may delay disease progression.
Wang et al. [47]	2016	Randomized Controlled Trial	33 patients (42 hips)	Harris Hip Score, Pain Score	Different doses of ESWT	Higher-dose ESWT produced superior pain relief and biological response.	Clinical response to ESWT appears dose-dependent.
Zhu et al. [48]	2023	Double-Blind Randomized Controlled Trial	72 patients	HHS, VAS, WOMAC	Individualized ESWT vs. celecoxib	ESWT produced greater pain relief and functional improvement.	ESWT is an effective non-operative treatment for symptomatic ONFH.
Dudani et al. [49]	2015	Retrospective Cohort Study	96 hips	Harris Hip Score	Bipolar hip arthroplasty	HHS improved from 39.3 to 89.1 at follow-up.	Bipolar arthroplasty provided satisfactory mid-term outcomes.
Gao et al. [50]	2015	Retrospective Case-Control Study	63 patients (79 hips)	Harris Hip Score	Cementless total hip arthroplasty	Significant functional improvement with satisfactory short-term survivorship.	Cementless THA is effective in extensive ONFH.

Assessment of Risk of Bias

Methodological quality of included randomized controlled trials demonstrated considerable variability when evaluated using the Cochrane RoB 2 framework [27]. Three studies were classified as having low overall RoB, reflecting appropriate randomization procedures, adequate management of missing data, and outcome assessment methods that were unlikely to introduce systematic error. The majority of the evidence base was judged to raise some concerns, most commonly due to incomplete reporting of allocation concealment procedures, limited information regarding adherence to assigned interventions, and insufficient detail to exclude the possibility of selective outcome reporting. Although these limitations do not necessarily invalidate the reported findings, they reduce confidence in the extent to which the observed treatment effects can be attributed solely to the interventions under investigation. Two studies were evaluated as having a high overall RoB because at minimum one critical domain was considered vulnerable to systematic error, particularly in relation to deviations from the expected interventions. Such methodological limitations might have influenced treatment exposure or participant behavior after randomization, thereby increasing the likelihood that the estimated intervention effects were affected by factors other than the assigned treatment. Collectively, an RoB assessment indicates that the current body of the evidence is characterized by predominantly moderate methodological certainty. While most studies employed randomized designs and reported clinically relevant outcomes, shortcomings in trial conduct and reporting warrant cautious interpretation of the findings and should be considered when concluding the effectiveness of interventions for ONFH, as summarized in Table 2.

**Table 2:** Risk-of-bias assessment of non-randomized interventions using the RoB 2 tool. RoB 2, Risk of Bias 2

Study	Bias arising from the randomization process	Bias due to deviations from intended interventions	Bias due to missing outcome data	Bias in the measurement of the outcome	Bias in the selection of the reported result	Overall risk of bias
Ma et al. (2014) [31]	Low risk	Low risk	Low risk	Low risk	Some concerns	Low risk
Li et al. (2016) [34]	Some concerns	Some concerns	Low risk	Low risk	Some concerns	Some concerns
Cao et al. (2017) [35]	Low risk	Some concerns	Some concerns	Low risk	Some concerns	Some concerns
Mao et al. (2015) [37]	Some concerns	Some concerns	Some concerns	Low risk	Some concerns	Some concerns
Pepke et al. (2016) [38]	Some concerns	Some concerns	Some concerns	Low risk	Some concerns	Some concerns
Xian et al. (2019) [40]	Some concerns	Some concerns	Low risk	Low risk	Some concerns	Some concerns
Aggarwal et al. (2020) [41]	Low risk	Low risk	Low risk	Low risk	Some concerns	Low risk
Hauzeur et al. (2020) [42]	Some concerns	Some concerns	Some concerns	Low risk	Some concerns	Some concerns
Lee et al. (2015) [44]	Low risk	High risk	Low risk	Low risk	Some concerns	High risk
Liyou et al. (2016) [45]	Some concerns	Some concerns	Low risk	Low risk	Some concerns	Some concerns
Camporesi et al. (2010) [46]	Low risk	Low risk	Some concerns	Low risk	Some concerns	Some concerns
Wang et al. (2016) [47]	Some concerns	High risk	Some concerns	Low risk	Some concerns	High risk
Zhu et al. (2023) [48]	Low risk	Low risk	Low risk	Low risk	Low risk	Low risk

Table 2 shows the RoB assessment, which demonstrated that most included studies had an overall judgment of either low risk or some concerns. The studies by Ma et al. [31], Aggarwal et al. [41], and Zhu et al. [48] were rated as having a low overall risk of bias. The study by Zhu et al. [48] was rated as having a low risk of bias across all domains, whereas the studies by Ma et al. [31] and Aggarwal et al. [41] raised some concerns in the reporting domain because of insufficient information regarding prespecified outcome reporting. Several studies were judged as having some concerns, primarily because of unclear randomization procedures, deviations from the intended interventions, incomplete outcome data, or insufficient reporting of outcomes [34,35,37,38,40,42,45,46]. High RoB was identified in the studies by Wang et al. [47] and Lee et al. [44], mainly due to significant deviations from intended interventions, which could have influenced the study results. Overall, measurement of outcomes was consistently assessed as low risk across all included studies, indicating reliable outcome assessment methods.

Observational Studies (NOS)

Among the observational studies, methodological quality ranged from poor to good [28]. Only three studies achieved a “good” overall rating, with scores ranging from 7 to 9, largely because their study designs included comparative control groups and utilized statistical matching or adjustments to account for baseline confounding factors [30,36,50]. The remaining six studies received a “poor” rating, with scores ranging from 5 to 7, primarily because they were observational case series or epidemiological profiles that either lacked an unexposed control group entirely or failed to adequately control for confounding variables. Under standard assessment thresholds, failure to control for confounders automatically results in zero stars for the Comparability domain, restricting the overall rating to “poor,” as shown in Table 3.

**Table 3:** Quality assessment for observational studies (Newcastle-Ottawa Scale). AVN, avascular necrosis; ARO, anterior rotational osteotomy; rhBMP-2, recombinant human bone morphogenetic protein-2; IBG, impacted bone grafting; CD, core decompression; CDBG, core decompression bone graft; CDSC, core decompression with stem cells.​​​​​​

Author (Study focus)	Selection (max 4)	Comparability (max 2)	Outcome (max 3)	Total score (0-9)	Overall rating
Vardhan et al. (Epidemiology) [29]	3	0	2	5	Poor
Lin et al. (Medial space ratio and collapse) [30]	4	1	2	7	Good
Elmalı et al. (Pedicled iliac graft) [32]	3	0	3	6	Poor
Sun et al. (rhBMP-2 vs. IBG) [33]	4	0	3	7	Poor
Kawano et al. (ARO vs. Conservative) [36]	4	2	3	9	Good
Talathi and Kamath (Stem cell implantation) [39]	3	0	3	6	Poor
Nally et al. (CD vs. CDBG vs. CDSC) [43]	4	0	3	7	Poor
Dudani et al. (Bipolar hip arthroplasty) [49]	3	0	3	6	Poor
Gao et al. (Arthroplasty in extensive AVN) [50]	4	2	3	9	Good

Discussion 

Interpretation of Results

The findings of the systematic review demonstrated that AVNFH is a multifactorial disease characterized by vascular insufficiency, impaired bone remodeling, and progressive structural deterioration of the femoral head. The epidemiological evidence identified corticosteroid exposure, alcohol consumption, trauma, and idiopathic causes as the predominant factors related to disease development, with corticosteroid use emerging as a highly reported risk factor [29]. These observations support the current understanding that disruption of femoral head microcirculation plays a central role in disease initiation and progression. The reviewed studies consistently indicated that the treatment outcomes are strongly influenced by disease stage at presentation. Joint-preserving interventions were associated with favorable outcomes, particularly in patients with pre-collapse disease, whereas advanced stages frequently required arthroplasty procedures [31-36]. Among surgical interventions, core decompression remained the most widely investigated procedure. Several included studies reported favorable clinical outcomes following core decompression; however, these findings should be interpreted cautiously because of differences in study design, sample size, interventions, and outcome measures. Superior outcomes were generally reported when biological augmentation strategies were incorporated. Bone marrow buffy coat grafting, peripheral blood stem-cell therapy, and platelet-rich plasma supplementation were associated with improved functional recovery, delayed radiographic progression, and enhanced hip survival compared with the conventional treatment [31,37,40,41]. Cell-based regenerative therapies were developed to address the impaired reparative capacity characteristic of osteonecrotic bone. Several studies reported promising outcomes with stem cell therapy, although the available evidence remains heterogeneous and should be interpreted cautiously, particularly when combined with mechanical decompression or structural support [31,37]. Nevertheless, not all regenerative approaches produced equivalent benefits. Bone marrow concentrate implantation and osteoblastic cell therapy generated only modest improvements, suggesting treatment efficacy might rely on cell preparation techniques, cellular composition, and disease characteristics [38,42]. Collectively, these findings indicate that regenerative medicine is becoming an important component of contemporary hip-preserving strategies. Rather than replacing conventional surgical procedures, biologic augmentation appears to enhance the reparative potential of core decompression by promoting angiogenesis, osteogenesis, and bone remodeling within the necrotic femoral head. Nevertheless, variations in cell processing techniques, biologic preparations, treatment protocols, and outcome measures across studies continue to limit direct comparison and widespread clinical implementation [39,40-43]. The evidence also highlighted the importance of restoring vascularity to the necrotic femoral head. Vascularized fibular grafting demonstrated superior femoral head perfusion, delayed disease progression, and improved hip survival when compared with core decompression alone [35]. Similar findings were reported following vascularized iliac bone grafting procedures [32]. These results reinforce the concept that revascularization remains a critical therapeutic target in AVNFH management. The available evidence also suggests that successful hip preservation depends on careful patient selection. Joint-preserving procedures consistently demonstrated more favorable outcomes in patients with precollapse disease, whereas the likelihood of treatment failure increased following femoral head collapse. These observations emphasize the importance of early diagnosis and timely intervention before irreversible structural deterioration occurs [31,32,35,36]. Among nonoperative interventions, hyperbaric oxygen therapy and ESWT produced encouraging outcomes. Hyperbaric oxygen therapy achieved long-term reduction in pain and functional improvement in patients with early-stage disease [46]. ESWT demonstrated dose-dependent clinical benefits and was associated to high pain relief and functional gains than pharmacological management with celecoxib [41,47]. These findings suggest selected nonoperative modalities may delay disease progression and reduce symptom burden in appropriately selected patients. Although these conservative interventions demonstrated encouraging short-term outcomes, they should be considered complementary rather than definitive treatments. Their role appears greatest in carefully selected patients with early-stage disease or in individuals who are unsuitable candidates for surgical intervention [46-48]. Pharmacological interventions demonstrated variable effectiveness. Intravenous Cervus and Cucumis polypeptides were associated with improvements in pain and hip function [45], whereas zoledronate therapy failed to significantly reduce femoral head collapse or the need for THA [44]. Collectively, available evidence suggests pharmacological treatment alone may be insufficient to modify the natural course of AVNFH. The inconsistent findings across pharmacological studies may reflect differences in disease stage at treatment initiation, duration of therapy, and the mechanisms of action of individual agents. Consequently, pharmacological management is currently best regarded as an adjunct to stage-appropriate surgical or regenerative treatment rather than a standalone therapeutic strategy [12-14,44,45]. For patients with advanced structural collapse and secondary degenerative changes, arthroplasty procedures remained the most predictable treatment option. Both bipolar hip arthroplasty and cementless THA produced substantial improvements in functional outcomes and patient satisfaction [49,50]. Findings indicate that although joint-preserving strategies are preferable in earlier stages, reconstructive procedures remain essential for advanced disease. These findings support a stage-based treatment strategy in which preservation of the native femoral head should be prioritized whenever feasible, while arthroplasty remains the preferred option for advanced disease associated with structural collapse and secondary degenerative changes [9,32,35,36,49,50].

Critical Limitations of the Evidence Base

Several limitations within the existing literature reduce the certainty of the available evidence. A major concern was the considerable heterogeneity among included studies. Variations were observed in patient populations, disease staging systems, intervention protocols, follow-up durations, and outcome measures. Such differences limit direct comparison between studies and complicate determination of the most effective treatment strategy. Sample size was another important limitation. Many studies enrolled relatively small patient cohorts, reducing statistical power and increasing the possibility of random error [35,38,42,46]. Small sample sizes also restrict the generalizability of findings to broader patient populations. Methodological quality varied substantially across studies. Although numerous randomized controlled trials were identified, several investigations employed retrospective or observational designs [29,32,36,39,43,47,49,50]. These study designs are more susceptible to selection bias, confounding variables, and incomplete data collection. The previously conducted RoB 2 assessment indicated that several studies presented methodological concerns related to randomization procedures, deviations from intended interventions, and selective reporting. Follow-up duration also differed considerably between studies. While some investigations reported long-term outcomes extending beyond five years [41,46], others evaluated only short-term clinical effects [38,48]. Consequently, the long-term durability of many interventions remains uncertain. Another limitation involved inconsistency in outcome assessment. Harris Hip Score was the most frequently reported measure; however, other studies utilized VAS, WOMAC, Lequesne Index, radiographic progression, hip survival rates, or conversion to THA [21-24,46,48]. This lack of standardization reduces comparability across studies and limits opportunities for quantitative synthesis.

Limitations of the Review Methodology

Several methodological limitations of the present systematic review should be acknowledged. First, the review included both randomized controlled trials and retrospective studies to provide a comprehensive overview of available evidence. Second, this approach was considered appropriate because high-quality randomized controlled trials are limited in AVNFH, particularly for surgical techniques, prognostic factors, and emerging biological therapies. Including observational studies and case series enabled a more comprehensive assessment of the available evidence; however, this also increased methodological heterogeneity, and the findings should therefore be interpreted cautiously. Third, outcome measures were not standardized across studies. Differences in functional scales, radiological assessments, and definitions of treatment success restricted direct comparison of results and prevented meaningful quantitative pooling of data. Fourth, publication bias cannot be excluded. Positive treatment outcomes are more likely to be published than negative findings, potentially resulting in an overestimation of intervention effectiveness. Finally, because of the pronounced clinical and methodological heterogeneity in various studies, meta-analysis was not feasible. Consequently, conclusions were derived from qualitative synthesis rather than pooled statistical estimates. A formal certainty of evidence assessment using the GRADE approach was not performed, which may limit the interpretation of the overall strength of the evidence.

Implications for Clinical Practice

The findings of this review support the importance of early diagnosis and stage-specific management in AVNFH. Patients diagnosed before femoral head collapse appear to derive the greatest benefit from joint-preserving interventions. Core decompression remains an appropriate first-line surgical option for early-stage disease; however, the evidence suggests that outcomes may be enhanced when biological augmentation techniques such as platelet-rich plasma, bone marrow buffy coat grafting, or stem-cell therapies are incorporated [31,37,40,41]. Vascularized grafting procedures may be considered in selected patients with larger lesions or higher risk of progression because of their ability to restore both structural support and vascularity [32,35]. Nonoperative modalities such as hyperbaric oxygen therapy and ESWT may serve as useful adjunctive treatments, particularly in patients who are poor surgical candidates or who present with pre-collapse disease [46-48]. For advanced stages characterized by femoral head collapse and secondary osteoarthritis, arthroplasty remains the most reliable intervention for pain relief and functional restoration [49,50].

Implications for Future Research

Future investigations should focus on large multi-center randomized controlled trials with adequate sample sizes and standardized outcome measures. Future research should also aim to establish standardized protocols for biologic augmentation, including optimal cell sources, processing methods, delivery techniques, and patient selection criteria. Greater consistency in these areas would improve comparability across studies and facilitate the translation of regenerative therapies into routine clinical practice. Such studies would improve comparability and strengthen the evidence base for treatment decision-making. Need for direct comparative trials evaluating emerging regenerative therapies, including platelet-rich plasma, bone marrow-derived cellular therapies, and stem-cell-based interventions, to determine their relative effectiveness and cost-effectiveness [31,37,38,40-42]. Long-term follow-up studies extending beyond five to 10 years are required to establish the durability of joint-preserving procedures and determine whether early clinical improvements translate into reduced rates of femoral head collapse and arthroplasty. Future research should also investigate prognostic factors that influence treatment success, including lesion size, disease stage, etiology, vascular status, and biomechanical parameters [29,30]. Improved patient stratification may facilitate the development of individualized treatment algorithms and optimize clinical outcomes. Finally, greater methodological consistency in reporting clinical outcomes, radiological progression, and adverse events would facilitate future evidence synthesis and allow more robust comparisons across studies.

## Conclusions

AVNFH is a progressive condition that primarily results from disruption of the blood supply, with corticosteroid use, excessive alcohol consumption, trauma, and idiopathic causes representing the most frequently reported risk factors. The evidence included in this review suggests that treatment outcomes are influenced by the stage of the disease. Early diagnosis, together with joint-preserving procedures such as core decompression, particularly when combined with biologic therapies, may provide better clinical outcomes before structural collapse of the femoral head occurs. Nonoperative interventions, including hyperbaric oxygen therapy and extracorporeal shock wave therapy, have shown potential for improving pain and functional status in selected patients, although the findings are not consistent across all studies. Pharmacological therapies demonstrated variable results, while THA remained the most commonly reported option for patients with advanced disease, offering consistent improvements in pain relief and functional recovery. Overall, the available evidence supports a stage-based approach to the management of AVNFH. However, the findings should be interpreted with caution because of the clinical and methodological heterogeneity of the included studies. Further well-designed prospective studies with standardized outcome measures and long-term follow-up are needed to strengthen the evidence base and guide future clinical practice.
